# Surveillance of Antibacterial Usage during the COVID-19 Pandemic in England, 2020

**DOI:** 10.3390/antibiotics10070841

**Published:** 2021-07-10

**Authors:** Amelia Andrews, Emma L. Budd, Aoife Hendrick, Diane Ashiru-Oredope, Elizabeth Beech, Susan Hopkins, Sarah Gerver, Berit Muller-Pebody, the AMU COVID-19 Stakeholder Group

**Affiliations:** 1 Healthcare-Associated Infection and Antimicrobial Resistance Division, Public Health England, London NW9 5EQ, UK; Emma.Budd@phe.gov.uk (E.L.B.); Aoife.Hendrick@phe.gov.uk (A.H.); Diane.Ashiru-Oredope@phe.gov.uk (D.A.-O.); Susan.Hopkins@phe.gov.uk (S.H.); Sarah.Gerver@phe.gov.uk (S.G.); Berit.Muller-Pebody@phe.gov.uk (B.M.-P.); 2 National Institute for Health Research, Health Protection Research Unit in Healthcare Associated Infections and Antimicrobial Resistance, University of Oxford, Oxford OX3 9DU, UK; 3 National Institute for Health Research, Health Protection Research Unit in Healthcare Associated Infections and Antimicrobial Resistance, Imperial College London, London W12 0NN, UK; 4 NHS England and NHS Improvement, London SE1 6LH, UK; Elizabeth.Beech@nhs.net

**Keywords:** antibacterials, antibiotics, COVID-19, antimicrobial stewardship

## Abstract

Changes in antibacterial prescribing during the COVID-19 pandemic were anticipated given that the clinical features of severe respiratory infection syndrome caused by SARS-CoV-2 mirror bacterial respiratory tract infections. Antibacterial consumption was measured in items/1000 population for primary care and in Defined Daily Doses (DDDs)/1000 admissions for secondary care in England from 2015 to October 2020. Interrupted time-series analyses were conducted to evaluate the effects of the pandemic on antibacterial consumption. In the community, the rate of antibacterial items prescribed decreased further in 2020 (by an extra 1.4% per month, 95% CI: −2.3 to −0.5) compared to before COVID-19. In hospitals, the volume of antibacterial use decreased during COVID-19 overall (−12.1% compared to pre-COVID, 95% CI: −19.1 to −4.4), although the rate of usage in hospitals increased steeply in April 2020. Use of antibacterials prescribed for respiratory infections and broad-spectrum antibacterials (predominately ‘Watch’ antibacterials in hospitals) increased in both settings. Overall volumes of antibacterial use at the beginning of the COVID-19 pandemic decreased in both primary and secondary settings, although there were increases in the rate of usage in hospitals in April 2020 and in specific antibacterials. This highlights the importance of antimicrobial stewardship during pandemics to ensure appropriate prescribing and avoid negative consequences on patient outcomes and antimicrobial resistance.

## 1. Introduction

In March 2020, the World Health Organization (WHO) declared the outbreak caused by the novel coronavirus SARS-CoV-2 a pandemic [1]. The high transmissibility of the virus and clinical severity of the associated coronavirus disease (COVID-19) have since challenged most aspects of healthcare delivery globally. This includes diagnosis, clinical management, and infection prevention and control measures related to COVID-19, but also the effective delivery of antimicrobial stewardship, particularly in early 2020 at the beginning of the pandemic [2].

Prior to the COVID-19 pandemic, total antibacterial consumption in England had been decreasing, mainly driven by reduced prescribing in primary care [3]. The decrease followed national awareness campaigns, changes in prescribing guidelines, and National Health Service (NHS) quality improvement and assurance schemes, all aimed at reducing prescribing in both primary and secondary care [3,4,5,6]. Changes in antibacterial prescribing patterns during the COVID-19 pandemic were anticipated given that clinical features of the severe respiratory infection caused by SARS-CoV-2 are similar to those caused by bacteria. Moreover, markers of infection such as C-reactive protein did not effectively distinguish between bacterial and viral pneumonia infections. Such diagnostic challenges complicated the decision for clinicians as to whether to prescribe antibiotics empirically [7].

In addition, other serious viral respiratory infections such as influenza are often complicated by bacterial and/or fungal co- or secondary infections [8,9]. Worldwide, the prevalence of co-infections in COVID-19 patients varies widely, from 0% to 46% in early studies [10], and the bacterial prevalence of both co/secondary infections was estimated to be 6.9% [11]. In England, co/secondary bacterial/fungal infections were infrequent (1%) among COVID-19 patients [12]. 

The International Severe Acute Respiratory and Emerging Infections Consortium reported that of 264,496 COVID-19 patients across 54 countries, 80% received antibiotics and 15% received antivirals. For patients admitted to intensive care units (ICU), an even higher percentage received antibiotics (93% of 24,822 patients) and antivirals (34% of 24,274 patients) [13]. In a rapid review, the prevalence of antibiotic prescribing among COVID-19 patients was 75%, with mechanical ventilation and older age associated with higher prescribing in COVID-19 patients [14]. Other studies have also shown high levels of empirical antibiotic treatment for hospitalised patients with COVID-19 in the early stages of the pandemic [15,16]. In primary care, a study from Scotland showed that the total number of prescriptions used for respiratory infections fell at the beginning of the pandemic [17].

To raise awareness of prudent antimicrobial use and guiding principles of antimicrobial stewardship, the WHO published interim guidance on the clinical management of COVID-19 in May 2020 [18]. It does not recommend antibiotic therapy or prophylaxis for patients with mild or moderate COVID-19 but advised empiric antibiotic treatment for patients with severe COVID-19, based on clinical judgement along with assessments for de-escalation. 

In England, the National Institute for Clinical Excellence (NICE) developed rapid clinical guidelines for COVID-19 including management of pneumonia (community and hospital) [7,19]. Across the UK, a third of organisations updated their local guidelines on community and hospital-acquired pneumonia based on the NICE national guidelines published in April 2020. Hospitals also reported a reduction in routine antimicrobial stewardship activities, with 64% reporting that COVID-19 had a negative impact on stewardship activities [20]. 

Concerns were raised that the COVID-19 pandemic would challenge the recent gains in prudent antibacterial use that protect patients from harm, such as *Clostridioides difficile* infections (CDI) following antibiotic treatment, and combat antimicrobial resistance [21,22]. We aim to describe the impact of the COVID-19 pandemic on antibacterial prescribing across primary and secondary care in England between January and October 2020, to inform antimicrobial stewardship activities during the ongoing COVID-19 challenges and future pandemic preparations.

## 2. Results

### 2.1. Primary Care

**Figure 1:**
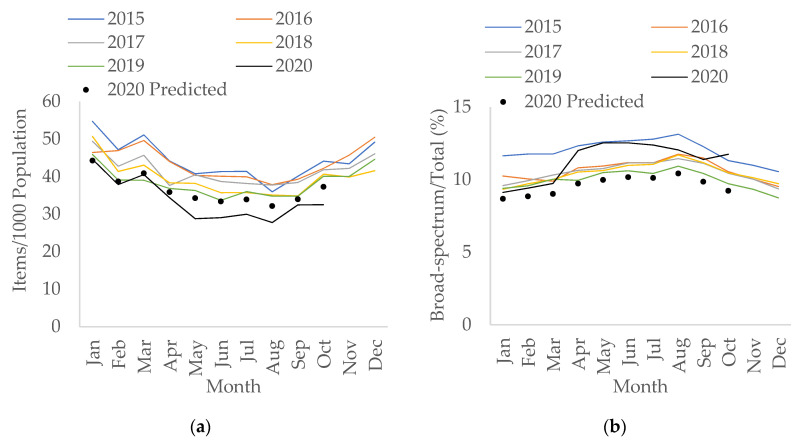
Interrupted time-series analysis for (**a**) total antibacterial items prescribed per 1000 population; (**b**) percentage of broad-spectrum antibacterials over total items prescribed, adjusted for seasonality, showing the counterfactual scenario (in black dots), in primary care, 2015–2020.

In England, total antibacterial prescribing in the community had been falling (by 0.3% per month; 95% confidence interval CI: −0.4 to −0.3, *p* < 0.05) prior to the pandemic. During the pandemic, it decreased by an additional 1.4% per month (95% CI: −2.3 to −0.5, *p* < 0.05). However, there was a slight increase in prescribing (by 1.5 items/1000 population) seen in March 2020 compared to March 2019 (Figure 1a).

Use of broad-spectrum antibacterials (co-amoxiclav, cephalosporins, and fluoroquinolones) in terms of items and as a percentage of total use in the community has also been decreasing since 2015. However, the percentage of broad-spectrum antibacterials over total prescriptions has changed to an upward trend during the pandemic with an increase of 0.2% per month (95% CI: 0.04 to 0.3, *p* < 0.05), as opposed to decreasing by 0.04% per month (95% CI: −0.04 to −0.03, *p* < 0.05) pre-COVID. The largest percentage increase was observed in April 2020 compared to April 2019 (by 20.6%) (Figure 1b). However, the increase in broad-spectrum items prescriptions during COVID-19 was not statistically significant (*p* = 0.111). This increase was mainly seen in April 2020 (Figure S1), with the increase from March to April 2020 (by 0.2 items/1000 population) mainly driven by the rise of co-amoxiclav, followed by cephalosporins; particularly first-generation cephalosporins (Supplementary Tables S1 and S2).

Antibacterials used for the treatment of community-acquired pneumonia (CAP) increased by 9.3% (2.2 items/1000 population) in March 2020 compared to March 2019 (Figure S2), although the increase in CAP prescriptions during COVID-19 overall was not statistically significant (*p* = 0.893). Specifically, prescriptions of oral amoxicillin and doxycycline increased by 4.1% and 28.9% (0.5 and 1.3 items/1000 population), respectively, in March 2020, compared to the same month in 2019. Amoxicillin prescriptions decreased sharply from March to May 2020 by 59.9% and remained low in the summer months. In contrast, there was a slight increase in doxycycline use in April 2020 compared to April 2019 (Figure S3).

### 2.2. Primary Care by Age Group

Prescriptions for which the age is unknown have decreased since becoming available in April 2015, from 7.3% to 1.1% in October 2020. Overall, total prescriptions for children (in the 0–4 and 5–14 age group) decreased during COVID-19 but the decreases were not statistically significant (*p* = 0.305 and 0.051, respectively). Most age groups saw a small increase in the number of total antibacterial items prescribed in March 2020 compared to March 2019, except for children below the age of 15. The slight increase seen in March 2020 was also observed for CAP prescriptions (Figure S4).

**Figure 2:**
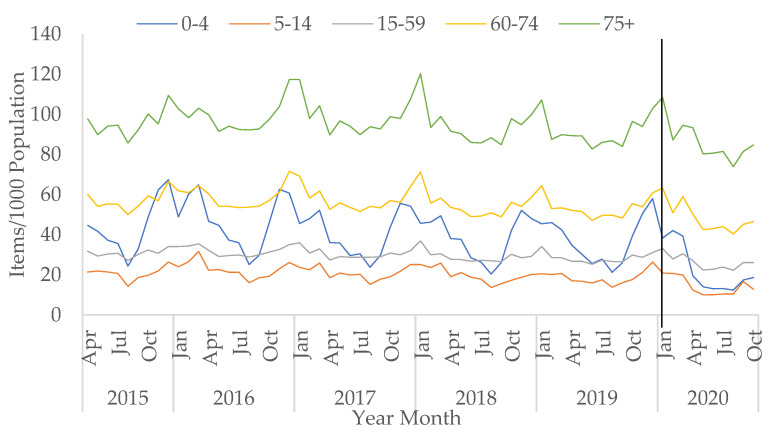
All antibacterial items prescribed per 1000 population in primary care by age group, April 2015–October 2020 (vertical black line represents the start of the COVID-19 pandemic in January 2020).

The rise in prescriptions in March 2020 compared to March 2019 was seen especially in older adults aged between 60 to 74, and over 75 (Figure 2). Prescriptions for broad-spectrum items in older adults over the age of 75 saw an increase in April 2020 compared to April 2019 (Figure S5). Specifically, a large rise was seen in co-amoxiclav prescribing for this age group between March and April 2020 by 24.8% (Figure S6).

In contrast, for infants and children up to four years, total prescriptions decreased sharply from March 2020 (−50.3% from March to April 2020) and remained low in the summer months (Figure 2). This age group had the largest decreasing trend in total prescriptions post-COVID-19 (by −6.1% per month, 95% CI: −8.1 to −4.1, *p* < 0.05). CAP prescriptions for infants and young children saw a large decrease between March and April 2020 (Figure S4), specifically amoxicillin prescriptions (−62.5%) (Figure S7).

### 2.3. Secondary Care

**Figure 3:**
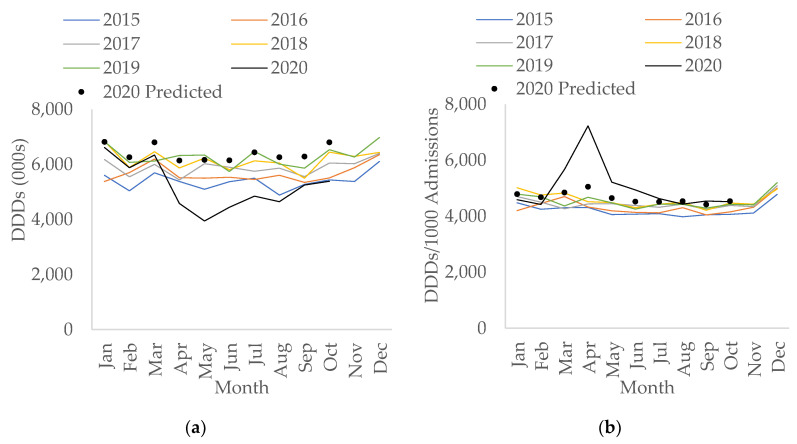
Interrupted time-series analysis for total antibacterial use in (**a**) Defined Daily Doses (DDDs); (**b**) DDDs per 1000 admissions, adjusted for seasonality, showing the counterfactual scenario (in black dots), in secondary care by month, 2015–2020.

In secondary care, the rate of total antibacterial consumption measured in Defined Daily Doses (DDDs)/1000 hospital admissions had been increasing year-on-year prior to COVID-19 (an increase of 0.2% per month, 95% CI: 0.09% to 0.3%, *p* < 0.05). During the first wave of the COVID-19 pandemic, the amount of total antibacterial use measured in DDDs has decreased to its lowest since 2015 (Figure 3a). The fall in total DDDs during COVID-19 overall was statistically significant (−12.1% compared to before COVID-19, 95% CI: −19.1 to −4.4, *p* < 0.05). However, when measuring total usage as the rate of antibacterial use per hospital admission, this increased overall during COVID-19 compared to before COVID-19 (by 12.0%, 95% CI: 2.6% to 22.3%, *p* < 0.05). Particularly, the rate doubled in April 2020 compared to April 2019 (7228 vs. 4681 DDDs/1000 admissions) and only returned to levels more in line with previous years from July 2020 onwards (Figure 3b). 

**Figure 4:**
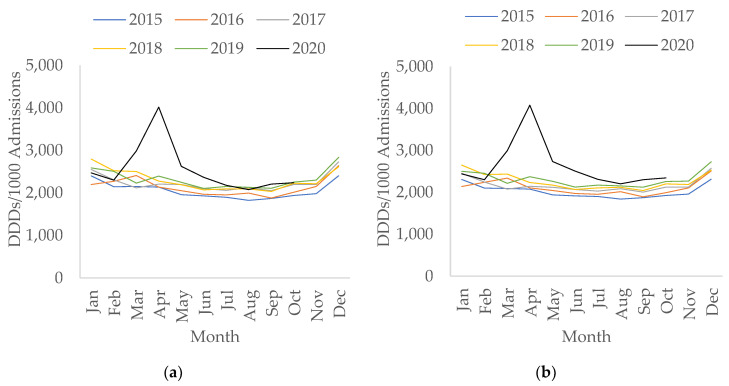
All antibacterials for treatment of (**a**) community-acquired pneumonia (CAP); (**b**) hospital-acquired pneumonia (HAP), per 1000 admissions in secondary care by month, 2015–2020.

The rate of antibacterials used for treatment of CAP and hospital-acquired pneumonia (HAP) in hospitals per 1000 admissions followed a similar trend to the rate of total antibacterial usage in hospitals—both saw overall increases during COVID-19 (*p* < 0.05) with a peak in usage seen in April 2020 (Figure 4).

**Figure 5:**
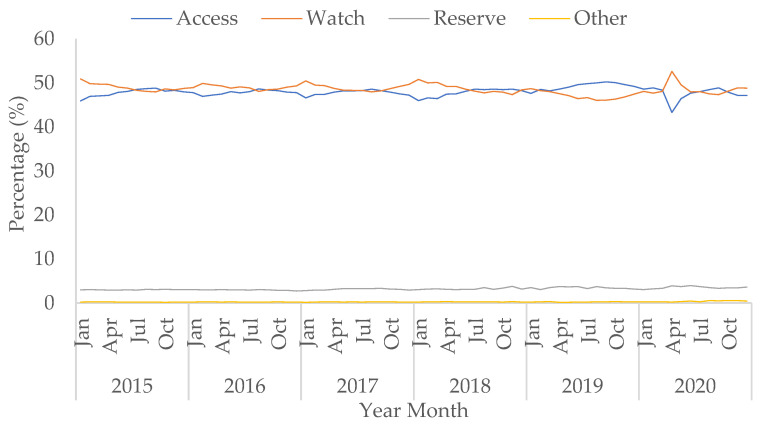
Percentage of prescribing in AWaRe antibacterial categories (from the WHO Essential Medicine List adopted for England) over total use in hospitals, January 2015–October 2020.

Although the use of doxycycline in the ‘Access’ group saw an increase of 59.5% in April 2020 compared to April 2019 (Figure S8), the percentage of all ‘Access’ antibacterial use decreased in April 2020 to 43.3% from 48.6% in April 2019. However, the decreasing trend in the percentage of ‘Access’ use during the pandemic was not statistically significant (*p* = 0.156). Usage of ‘Access’ antimicrobials then increased to 48.5% in August 2020, similar to pre-pandemic levels (Table S3). Conversely, the percentage of ‘Watch’ and ‘Reserve’ antibacterial use increased during COVID-19, especially in April 2020. The increasing trends of the percentage of ‘Watch’ and ‘Reserve’ use during COVID-19 were not statistically significant (*p* = 0.374 and 0.373, respectively), and the percentage use for both then decreased to similar levels observed prior to the peak of the first wave in July and August (Figure 5).

The rise in use of ‘Watch’ category antibacterials was mainly due to the increased use of co-amoxiclav, third-generation cephalosporins (specifically ceftriaxone), and macrolides (Figures S9 and S10). The rise in macrolide usage was mainly driven by clarithromycin, with small increases seen in azithromycin and erythromycin (Figure S10). The rate of piperacillin/tazobactam use within the ‘Watch’ category increased by 82.8% in April 2020 compared to April 2019. This baseline level is historically low following a global shortage in 2017 (Figure S11). The rise in the percentage of ‘Reserve’ category antibiotics by 0.2 percentage points in April 2020 in comparison to April 2019 was predominantly due to a 79.5% increase in the use of carbapenems (Figure S12).

## 3. Discussion

This study is the first to describe national antibacterial use in both primary and secondary care during the COVID-19 pandemic between January and October 2020. In England, total antibacterial use measured in items and DDDs in primary and secondary care, respectively, reduced overall during the first wave of the pandemic. However, the rate of antibacterial usage by hospital admission increased steeply in March and April 2020, despite low rates of bacterial co-infections being reported for COVID-19 patients [10,11,23]. This likely reflects unfamiliarity with treating a new pathogen, and uncertainties around bacterial co-infection and secondary infection at the time. Moreover, the rate of total prescribing in the community was lower than in previous years from April to October 2020, also seen at the beginning of the pandemic in British Columbia, Canada [24]. This probably reflects changes in healthcare-seeking behaviour, access to healthcare, and reduced transmission of other infectious pathogens due to the adoption of population-level measures to reduce transmission of SARS-CoV-2 in the community (‘lockdown’) [25].

In the community, total and broad-spectrum prescribing has been falling since 2014 following the introduction of NHS quality improvement initiatives and awareness campaigns [3,26,27]. However, from April 2020 onwards the percentage of total items comprised of broad-spectrum antibacterials increased. This is partly due to the large decrease in prescriptions of the narrow-spectrum amoxicillin for infants and young children. During the same time period, respiratory tract infections also decreased significantly, likely due to reduced mixing of children from the closure of schools and early years settings during the community lockdown [28].

With COVID-19 changing much of prescribing practice and healthcare-seeking behaviour by patients, comparisons of percentages of total prescriptions should be interpreted with caution. Nonetheless, a small rise in the number of broad-spectrum antibacterial items was seen in April 2020 compared to April 2019, particularly in older adults over the age of 75, driven by co-amoxiclav prescribing in this age group. The co-amoxiclav increase seen in older patients was also observed in North West London [29]. This may reflect treatment of other primary infections, such as urinary tract infections, to avoid hospital admission during the pandemic, further work is required to evidence whether the antibacterial use was appropriate. The use of certain broad-spectrum antibacterials, particularly cephalosporins and quinolones, is associated with increased risks of CDI and should be reserved for treating resistant infections [22]. Although the count of CDI in April to June 2020 decreased compared to the same period in the previous year, reported community-onset, community-associated CDI increased slightly [30]. Further investigations are needed to understand the large reduction of antibacterial use in the community and its impacts on infections.

At the beginning of the COVID-19 pandemic, more antibacterial items for respiratory infections were prescribed in primary care in England in March 2020 when compared to March 2019. This was also seen in Scotland [17] and is thought to be due to additional prescribing of ‘just in case’ rescue antibiotic packs for chronic obstructive pulmonary disease patients at risk of severe respiratory disease. Oral amoxicillin and oral doxycycline were recommended as first-choice treatments in the pre-COVID NICE community-acquired pneumonia guideline [31], and the usage of both antibacterials increased in March 2020 compared to March 2019. Both antibiotics were commonly prescribed within 14 days of a positive SARS-CoV-2 test in North West London [29]. While amoxicillin use saw a sharp decrease from March to May 2020, doxycycline use increased slightly in April 2020 compared to April 2019 when the COVID-19 rapid guideline was published, as doxycycline was recommended over amoxicillin due to the broader spectrum of cover against secondary bacterial causes of pneumonia [19]. 

The reduction in antibiotic items prescribed in the community may be related to ‘lockdown’ and the fall in general practice (GP) attendance (face-to-face, virtual and telephone included) in March 2020 in England [32]. The majority of antibacterial prescribing in primary care is for respiratory or urinary tract infections [33] and the consultation rates of both infections have decreased in the UK during the pandemic [34]. There was also a change in GP appointment mode from a majority of face-to face to telephone/virtual appointments [32]. The high percentage of broad-spectrum prescribing from total items in both in-hours and out-of-hours GP, especially for co-amoxiclav and doxycycline in out-of-hours between March and May 2020 [35], may reflect higher levels of “precautionary” antibacterial prescribing in remote consultations compared to in-person appointments. 

In hospitals, the total volume of antibacterial use measured in DDDs fell sharply in April 2020 and remains lower than levels seen in the preceding five years. However, the rate of prescribing measured in DDDs/1000 admissions increased sharply in April 2020, only returning in July 2020 to similar levels seen in previous years. This is likely to be due to hospitals implementing rapid changes in healthcare service provision such as cancellation of elective admissions and staff redeployment in March 2020 to adapt hospital capacity to COVID-19 treatment demands [36]. Hence, although hospital activity was greatly diminished during the first wave of the pandemic, patients who were admitted to hospital were likely to be more acutely and seriously ill and their clinical treatment potentially more driven by ‘therapeutic aggressiveness’ in situations with sometimes scarce evidence [37]. A more granular measure of hospital activity during the pandemic is required to better understand the effect of COVID-19 on antibacterial prescribing in secondary care. 

Our data show that antibacterials classified to the ‘Watch’ (mainly broad-spectrum antibacterials) or ‘Reserve’ (‘last-resort’ or new antibacterials) categories using the WHO’s AWaRe index observed increases during the pandemic. Another study has also shown that broad-spectrum antibiotic use in hospitals was common [38], despite insufficient evidence of a high percentage of hospitalised COVID-19 patients with bacterial co-infection [10,11]. Third-generation cephalosporins, part of the ‘Watch’ category, doubled in use in April 2020 compared to April 2019; with specific increases seen in ceftriaxone. This is thought to be due to Trusts selecting the once daily ceftriaxone to save nursing time. However, the percentage of ‘Watch’ use we present is lower than shown in other studies [11]. Conversely, despite the ‘Access’ category containing antibacterials that are recommended as first-line treatments for CAP and HAP, the percentage of their use was lower during the first wave of the pandemic. This may be due to approximately 40% of hospitals in the UK reporting that they were already aligned with the published national guidelines, while 12% stated they did not plan to update their local guidelines [20]. 

Doxycycline and azithromycin were hypothesised as treatments for COVID-19 due to their anti-inflammatory properties and are being investigated in clinical trials in primary and secondary care, respectively. Preliminary results show no benefit of azithromycin in hospitalised patients with COVID-19 and no benefit of either antibiotic for early stages COVID-19 patients over the age of 50 in the community [39,40,41]. WHO does not recommend use of azithromycin as treatment of COVID-19 outside of clinical trials due to cardiotoxicity concerns [18]. Our data showed that azithromycin usage in hospital in April 2020 has increased but only slightly, despite considerable media attention given to the potential therapeutic role of azithromycin. This is encouraging and suggests it was not widely used outside trial settings. Although increases in oral doxycycline use occurred prior to the publication of the COVID-19 rapid guidelines, this antibacterial was included in all the national pneumonia guidelines before and during the pandemic. 

Continued enhanced surveillance is required, as inappropriate antibacterial use could lead to long-term unintended consequences on antimicrobial resistance, and potentially adverse outcomes for patients. In future waves of COVID-19, effective antimicrobial stewardship is required, especially given reported reductions in routine antimicrobial stewardship activities across hospitals in the UK during the first wave of the pandemic [20]. This is to prevent the hard-won gains from previous NHS stewardship schemes, such as the Commissioning for Quality and Innovation scheme [6,26], from being jeopardised. As the pandemic reached different areas of England at different times from September 2020 [42], more work is required to understand the impact of COVID-19 on antimicrobial prescribing during subsequent waves, bearing in mind antibacterial use in winter is generally high for respiratory infections. Additionally, the impact of COVID-19 on antimicrobial prescribing at a regional level as waves were felt differentially across the country.

To fully understand the impact of COVID-19 on antimicrobial use, patient-level prescribing data, including indications, is required, especially as no demographic data were available for secondary care nationally. This would allow the impact of antimicrobial treatment, including antifungals, on serious respiratory tract infections to be studied by linking to patient outcomes and laboratory records for antimicrobial susceptibility testing results. Moreover, since healthcare provision and healthcare-seeking behaviour changed significantly during the first months of the pandemic, additional information on primary care consultations are needed to interpret and adjust for changes in prescribing rates. The role of inflammatory markers needs to be investigated in research such as the National Institute for Health Research funded PEACH trial for procalcitonin [43], as there is currently limited evidence for their routine use but they have been indicated by hospitals to guide decisions for antibacterial treatments [7]. In addition, further work is needed to monitor antimicrobial use for long-term sequelae of COVID-19, alongside potential unintended consequences of changes in antibiotic use on increasing antimicrobial resistance.

## 4. Materials and Methods

### 4.1. Data Sources

Clinical commissioning groups (CCGs) are groups of general practices (GP) commissioned for primary care services in their local areas in England. Primary care prescribing included all antibacterial drugs (within British National Formulary chapter 5.1) for all CCGs from dispensed NHS prescriptions in the community, including general practice, out-of-hours, and urgent care. Monthly data were extracted from January 2015 to October 2020 (via ePACT2 from the NHS Business Services Authority) [44]. Data were also extracted by five-year age groups from April 2015. Mid-year CCG population data were obtained from the Office for National Statistics, with the population for 2019 used as a proxy for 2020. 

NHS acute Trusts are organisations comprising groups of NHS hospitals under the same management that are commissioned to provide secondary healthcare. For secondary care, monthly antibacterial data in Defined Daily Doses (DDDs) for all NHS acute Trusts in England were sourced from Rx-info (Define). Data were extracted from January 2015 to October 2020 using the WHO 2019 DDD index [45]. Hospital admissions data for Trusts were obtained from Hospital Episode Statistics sourced from NHS Digital, with 2020/21 admissions data being provisional.

### 4.2. Descriptive and Statistical Analysis

Antibacterial consumption was measured in antibacterial items/1000 population in primary care and in DDDs/1000 hospital admissions for secondary care. The trends and changes in consumption from 2015 to October 2020 were described for antibacterials listed in Section 4.3. 

Interrupted time-series analyses were conducted to evaluate changes in antibacterial consumption in England from the COVID-19 pandemic. This is a quasi-experimental design that can be used to assess whether changes from the population-level interventions are greater than the underlying trend before the intervention [46]. The ‘first-wave’ of SARS-CoV-2 infections in England was defined for the purpose of this study as January 2020 to October 2020 (the ‘intervention’). The pre-pandemic period was defined from January 2015 (April 2015 for primary care age group analysis) to December 2019. The dummy intervention variable was set to 0 pre-COVID and 1 post-COVID.

Monthly time-series were constructed using antibacterial consumption as the outcome variable for the pre- and post-COVID periods. Negative binominal regression models (maximum likelihood time-series analysis) were fitted for the event count data according to the distribution of the outcome [47,48]. Seasonality was adjusted for (in calendar months) as an independent variable in the model, resulting in incidence-rate ratios with 95% confidence intervals [48]. The percentages of specific antibacterials to total prescriptions before and after COVID-19 were compared using linear regression. The regression models were used to predict antibacterial consumption during COVID-19 that would be expected in the absence of the pandemic using the underlying trend (the counterfactual scenario). For the equations and results of the regression models, see Tables S4 and S5. 

Stata 15 was used in all data analyses [49].

### 4.3. Antibacterials Selection

Selection of antibacterials was based on routinely monitored groups under surveillance by Public Health England and published on the Fingertips portal [50], the annual English surveillance programme for antimicrobial utilisation and resistance (ESPAUR) Report [3], NHS improvement and assurance schemes [5,6], and treatment guidelines published by NICE, including:

- Total antibacterial use in both primary and secondary care settings;
- ‘Broad-spectrum antibacterials’, which included amoxicillin/clavulanic acid (co-amoxiclav), cephalosporins, and fluoroquinolones for primary care;
- AWaRe categories from the WHO Essential Medicine List adopted in England for hospital settings: antibacterials to improve access to (Access, predominantly narrow-spectrum), to monitor (Watch, predominantly broad-spectrum), and for ‘last resort’ or new antibacterials (Reserve) [51];
- Antibacterials for treatment of pneumonia; community-acquired pneumonia (CAP) and hospital-associated pneumonia (HAP) now for children and young people only [31,52], and secondary pneumonia with COVID-19 infection for adults [7,19], were defined using the NICE guidelines and the UK Advisory Committee on Antimicrobial Prescribing and Resistance and Healthcare Associated Infection survey (personal communication);
- Antibacterials for treatment of respiratory tract infections (besides tuberculosis) including treatments for ventilator-associated Gram-negative infections;
- Specific groups of antibacterials were also investigated; second- and third-generation cephalosporins for treatment of secondary bacterial infections and macrolide antibacterials undergoing clinical trials in hospitals; azithromycin for hospital inpatients in RECOVERY [53], and macrolides for ICU patients in REMAP-CAP [54].

A full list of antibacterials including their Anatomical Therapeutic Chemical codes is available in Table S6 for CAP and HAP, and Table S7 for all other selected antibacterials.

## 5. Conclusions

In conclusion, overall volumes of antibacterials prescribed at the beginning of the COVID-19 pandemic in 2020 decreased in both primary and secondary care in England. However, antibacterial usage per hospital admission increased steeply in April 2020 due to changes in the hospital population. Use of antibacterials prescribed for respiratory infections and broad-spectrum antibacterials increased in both settings. This highlights the urgent need for antimicrobial stewardship to ensure appropriate prescribing and avoid negative consequences on patient outcomes and antimicrobial resistance.

## Data Availability

The data presented in this study are available in the article and the supplementary materials. All data sources have been referenced in the study.

## Supplementary Materials

The following are available online at https://www.mdpi.com/article/10.3390/antibiotics10070841/s1, Table S1: Broad-spectrum items prescribed per 1000 population in primary care, December 2019–October 2020. Table S2: Cephalosporins items prescribed per 1000 population in primary care, December 2019–October 2020. Table S3: Percentage of AWaRe antibacterial categories over total antibacterial consumption in DDDs per 1000 admissions in hospitals, April 2019–October 2020. Table S4: Results of the interrupted time-series analyses using negative binomial regression with incidence rate ratios (IRR) and 95% confidence intervals (CIs). Table S5: Results of the interrupted time-series analyses using linear regression with coefficients and 95% confidence intervals. Table S6: Antibacterial groups with ATC codes for community-acquired pneumonia (CAP) and hospital-acquired pneumonia (HAP). Table S7: Antibacterial groups with ATC codes for all other selected antibacterials. Figure S1: All broad-spectrum items prescribed per 1000 population in primary care, January 2015–October 2020. Figure S2: All antibacterial items for treatment of community-acquired pneumonia per 1000 population in primary care, January 2015–October 2020. Figure S3: First-line antibacterial items recommended for treatment of community-acquired pneumonia per 1000 population in primary care, January 2015–October 2020. Figure S4: All antibacterial items for treatment of community-acquired pneumonia per 1000 population in primary care by age group, April 2015–October 2020. Figure S5: All broad-spectrum items prescribed per 1000 population in primary care by age group, April 2015–October 2020. Figure S6: Co-amoxiclav items prescribed per 1000 population in primary care by age group, April 2015–October 2020. Figure S7: Oral amoxicillin items per 1000 population in primary care by age group, January 2015–October 2020. Figure S8: Oral amoxicillin and oral doxycycline use in DDDs per 1000 admissions in secondary care, January 2015–October 2020. Figure S9: Third-generation cephalosporin use in DDDs per 1000 admissions in secondary care, January 2015–October 2020. Figure S10: Macrolides use in DDDs per 1000 admissions in secondary care, January 2015–October 2020. Figure S11: Piperacillin/tazobactam use in DDDs per 1000 admissions in secondary care, January 2015–October 2020. Figure S12: Carbapenems use in DDDs per 1000 admissions in secondary care by month, January 2015–October 2020.
