# Supplementary material for: Surveillance of Antibacterial Usage during the COVID-19 Pandemic in England, 2020
Source: Antibiotics (Basel). 2021 Jul 10;10(7):841. doi: 10.3390/antibiotics10070841 (PMC8300678; doi:10.3390/antibiotics10070841)
Supplement: Supplementary file 1 [file antibiotics-10-00841-s001.zip › antibiotics-1219126-supplementary.pdf]

Supplementary data

Table S1. Broad-spectrum items prescribed per 1000 population in primary care, December 2019 – October 2020

Month-year	Co-amoxiclav	Cephalosporins	Fluoroquinolones	Broad-spectrum Total
Dec-19	1.97	1.15	0.79	3.90
Jan-20	2.04	1.19	0.84	4.07
Feb-20	1.77	1.08	0.72	3.57
Mar-20	1.97	1.19	0.79	3.95
Apr-20	2.11	1.23	0.80	4.13
May-20	1.76	1.12	0.72	3.61
Jun-20	1.76	1.15	0.74	3.64
Jul-20	1.76	1.19	0.76	3.71
Aug-20	1.59	1.09	0.67	3.35
Sep-20	1.76	1.20	0.75	3.70
Oct-20	1.79	1.24	0.79	3.82

Table S2. Cephalosporins items prescribed per 1000 population in primary care, December 2019 – October 2020

	Cephalosporins		
**Month-year**	**First-generation**	**Second-generation**	**Third-generation**
Dec-19	1.125	0.019	0.004
Jan-20	1.167	0.020	0.005
Feb-20	1.055	0.016	0.004
Mar-20	1.168	0.019	0.004
Apr-20	1.208	0.017	0.003
May-20	1.104	0.015	0.004

Jun-20	1.133	0.013	0.004
Jul-20	1.176	0.014	0.004
Aug-20	1.072	0.012	0.003
Sep-20	1.181	0.014	0.004
Oct-20	1.219	0.014	0.007

Table S3. Percentage of AWaRe antibacterials categories over total antibacterial consumption in DDDs per 1000 admissions in hospitals, April 2019 – October 2020

Month-year	% Access	% Watch	% Reserve	% Other
Apr-19	48.55	47.57	3.71	0.17
May-19	49.05	47.10	3.69	0.16
Jun-19	49.59	46.44	3.73	0.23
Jul-19	49.82	46.66	3.29	0.22
Aug-19	49.98	46.02	3.72	0.27
Sep-19	50.22	46.10	3.44	0.24
Oct-19	50.05	46.32	3.33	0.29
Nov-19	49.59	46.83	3.32	0.27
Dec-19	49.20	47.42	3.13	0.24
Jan-20	48.59	48.08	3.04	0.28
Feb-20	48.83	47.69	3.21	0.27
Mar-20	48.37	48.04	3.31	0.28
Apr-20	43.27	52.59	3.90	0.23
May-20	46.44	49.53	3.74	0.30
Jun-20	47.67	47.95	3.95	0.42
Jul-20	47.99	48.02	3.70	0.29
Aug-20	48.48	47.49	3.49	0.54
Sep-20	48.88	47.33	3.30	0.48

Oct-20	47.92	48.10	3.42	0.56

Table S4. Results of the interrupted time-series analyses using negative binomial regression^ with incidence rate ratios (IRR) and 95% confidence intervals (CIs)

	**Pre-COVID-19**		**Post-COVID-19**			
	**Trend pre-COVID**		**COVID impact overall**		**Trend post-COVID**	
**Antibacterials**	IRR	95% CI	IRR	95% CI	IRR	95% CI
**Primary care**						
Total	-0.3%*	-0.4 to -0.3	-0.4%	-6.0 to 5.6	-1.7%*	-2.6 to -0.9
Broad-spectrum	-0.6%*	-0.7 to -0.6	5.2%	-1.2 to 12.0	-0.3%	-1.3 to 0.6
CAP	-0.4%*	-0.5 to -0.3	0.6%	-8.1 to 10.1	-3.4%*	-4.7 to -2.1
**Primary care by age group**						
0-4	-0.6%*	-0.7 to -0.4	-7.2%	-19.6 to 7.1	-6.1%*	-8.1 to -4.1
5-14	-0.4%*	-0.6 to -0.3	-13.7%	-25.6 to 0.07	-1.5%	-3.7 to 0.7
15-69	-0.3%*	-0.3 to -0.2	1.0%	-4.6 to 7.0	-1.2%*	-2.1 to -0.4
60-74	-0.2%*	-0.3 to -0.2	2.1%	-3.8 to 8.3	-1.8%*	-2.6 to -0.9
75+	-0.2%*	-0.2 to -0.1	2.8%	-3.0 to 8.8	-1.3%*	-2.2 to -0.4
**Secondary care**						
Total (DDDs only)	0.3%*	0.2 to 0.4	-12.1%*	-19.1 to -4.4	-1.6%*	-2.8 to -0.4
Total (DDDs/admissions)	0.2%*	0.09 to 0.3	12.0%*	2.6 to 22.3	-0.6%	-1.9 to 0.8
CAP	0.3%*	0.2 to 0.4	12.5%*	0.9 to 25.3	-0.8%	-2.4 to 0.8
HAP	0.3%*	0.2 to 0.4	14.5%*	3.1 to 27.1	-0.4%	-2.0 to 1.2

^ Equation for negative binomial regression:

$$\text{Log}(Y_t) = \beta_0 + \alpha(m_t) + \beta_1 X_t + \beta_2 T_t + \beta_3 X_t T_t + \log(N_t)$$

Where

Y_t is the dependent variable with monthly number of specific antibacterials

$\alpha(m_t)$ is the seasonal effect of month *m* at time *t*

X_t is a dummy variable representing COVID-19 (pre-COVID is 0 and post-COVID is 1). For the counterfactual scenario, $X_t = 0$.

T_t is the time (in months) centred around COVID-19

β_0 is the intercept (log rate at *t*=0)

β_1 is the change in base rate at the time of COVID-19

β_2 is the trend in the pre-COVID period

β_3 is the change in trend in the COVID-19 period

N_t is the population size at time t

* $p<0.05$

Table S5. Results of the interrupted time-series analyses using linear regression^ with coefficients and 95% confidence intervals (CIs)

	**Pre-COVID-19**		**Post-COVID-19**			
	**Trend pre-COVID**		**COVID impact overall**		**Trend post-COVID**	
**Antibacterials**	Coefficient	95% CI	Coefficient	95% CI	Coefficient	95% CI
**Primary care – Broad-spectrum/Total**						
% Broad-spectrum	-0.04%*	-0.04 to -0.03	0.6%	-0.1 to 1.4	0.2%*	0.04 to 0.3
**Secondary care – AWaRe categories/Total**						
% Access	0.02%*	0.01 to 0.4	-0.3%	-1.6 to 1.1	-0.1%	-0.3 to -0.06
% Watch	-0.03%*	-0.05 to -0.02	0.4%	-0.9 to 1.8	0.09%	-0.1 to 0.3
% Reserve	0.01%*	0.007 to 0.01	-0.05%	-3.6 to 0.3	0.02%	-0.03 to 0.07
% Other	0.0007%*	0.00008 to 0.01	-0.08%*	-0.1 to -0.02	0.03%*	-0.02 to 0.4

^ Equation for linear regression:

$$Y_t = \beta_0 + \alpha(m_t) + \beta_1 X_t + \beta_2 T_t + \beta_3 X_t T_t + e_t$$

Where

Y_t is the dependent variable with monthly percentage of specific antibacterials over total prescriptions

$\alpha(m_t)$ is the seasonal effect of month *m* at time *t*

X_t is a dummy variable representing COVID-19 (pre-COVID is 0 and post-COVID is 1). For the counterfactual scenario, $X_t = 0$.

T_t is the time (in months) centred around COVID-19

β_0 is the intercept (percentage at t=0)

β_1 is the change in percentage at the time of COVID-19

β_2 is the trend in the pre-COVID period

β_3 is the change in trend in the COVID-19 period

e is random error

* $p<0.05$

Table S6. Antibacterial groups with ATC codes for community-acquired pneumonia (CAP) and hospital-acquired pneumonia (HAP)

			CAP Guidelines			HAP Guidelines		
**Antibacterial Group**	ATC code	ATC Name (route#)	COVID-19 rapid NICE [1]	NICE [2]	APRHAI survey*	COVID-19 rapid NICE [3]	NICE [4]	APRHAI survey*
Tetracyclines	J01AA01	Demeclocycline						
	J01AA02	Doxycycline (O)	Y	Y	Y	Y	Y	Y
		Doxycycline (P)						
	J01AA04	Lymecycline						
	J01AA06	Oxytetracycline						
	J01AA07	Tetracycline						
	J01AA08	Minocycline						
	J01AA12	Tigecycline						
Amphenicols	J01BA01	Chloramphenicol						
Penicillin (excluding inhibitors)	J01CA01	Ampicillin						
	J01CA04	Amoxicillin (O)	Y	Y				
		Amoxicillin (P)		Y	Y			
	J01CA08	Pivmecillinam						
	J01CA12	Piperacillin						
	J01CA17	Temocillin						
	J01CA51	Ampicillin, combinations						
	J01CE01	Benzylpenicillin						
	J01CE02	Phenoxymethylpenicillin						
	J01CF05	Flucloxacillin						
Penicillin (with inhibitor combinations)	J01CR02	Amoxicillin and beta-lactamase inhibitor (O)		Y	Y	Y	Y	
		Amoxicillin and beta-lactamase inhibitor (P)			Y			

	J01CR03	Ticarcillin and beta-lactamase inhibitor						
	J01CR05	Piperacillin and beta-lactamase inhibitor				Y	Y	Y
	J01CR50	Combinations of penicillins						
First-generation cephalosporins	J01DB01	Cefalexin					Y	
	J01DB04	Cefazolin						
	J01DB05	Cefadroxil						
	J01DB09	Cefradine						
Second-generation cephalosporins	J01DC01	Cefoxitin						
	J01DC02	Cefuroxime (O)						
		Cefuroxime (P)					Y	
	J01DC04	Cefaclor						
Third-generation cephalosporins	J01DD01	Cefotaxime						
	J01DD02	Ceftazidime				Y	Y	
	J01DD04	Ceftriaxone		Y	Y		Y	
	J01DD08	Cefixime						
	J01DD13	Cefpodoxime						
	J01DD52	Ceftazidime and beta-lactamase inhibitor					Y	
Fourth-generation cephalosporins	J01DE01	Cefepime						
Monobactams	J01DF01	Aztreonam						
Carbapenems	J01DH02	Meropenem					Y	
	J01DH03	Ertapenem						
	J01DH04	Doripenem						
	J01DH51	Imipenem/cilastatin						
Fifth-generation cephalosporins	J01DI01	Ceftobiprole medocaril						
	J01DI02	Ceftaroline fosamil						

	J01DI54	Ceftolozane and beta-lactamase inhibitor						
Sulfonamides and trimethoprim	J01EA01	Trimethoprim						
	J01EB04	Sulfapyridine						
	J01EC02	Sulfadiazine						
	J01ED05	Sulfamethoxypyridazine						
	J01EE01	Co-trimoxazole				Y	Y	
Macrolides	J01FA01	Erythromycin		Y	Y			
	J01FA02	Spiramycin			Y			
	J01FA09	Clarithromycin		Y	Y		Children	
	J01FA10	Azithromycin (O & P)			Y			
	J01FA15	Telithromycin			Y			
Lincosamides	J01FF01	Clindamycin						
Streptogramins	J01FG01	Pristinamycin						
	J01FG02	Quinupristin/dalfopristin						
Aminoglycosides	J01GB01	Tobramycin						
	J01GB03	Gentamicin						
	J01GB05	Neomycin						
	J01GB06	Amikacin						
Fluoroquinolones	J01MA01	Ofloxacin						
	J01MA02	Ciprofloxacin						
	J01MA06	Norfloxacin						
	J01MA12	Levofloxacin (O & P)		Y		Y	Y	
	J01MA14	Moxifloxacin						
Other quinolones	J01MB02	Nalidixic acid						
Glycopeptides	J01XA01	Vancomycin						
	J01XA02	Teicoplanin						
Polymyxins	J01XB01	Colistin						

Steroid antimicrobials	J01XC01	Fusidic acid						
Imidazole derivatives	J01XD01	Metronidazole						
	J01XD02	Tinidazole						
Nitrofuran derivatives	J01XE01	Nitrofurantoin						
Other antibacterials	J01XX01	Fosfomycin						
	J01XX05	Methenamine						
	J01XX08	Linezolid						
	J01XX09	Daptomycin						
	J01XX11	Tedizolid						
Oral Metronidazole	P01AB01	Metronidazole						
Anti-*Clostridioides difficile* agents	A07AA09	Vancomycin						
	A07AA12	Fidaxomicin						

[#] Route of administration: O = Oral, P = Parenteral

The COVID-19 rapid National Institute for Health and Care Excellence (NICE) guidelines are for adults only and the previous NICE guidelines are for children and young people only during the COVID-19 pandemic

* Advisory Committee on Antimicrobial Prescribing and Resistance and Healthcare Associated Infection (APRHAI) survey was a personal communication

Table S7. Antibacterial groups with ATC codes for all other selected antibacterials

			**Clinical Trials**		**Primary care**	**Secondary care**	
**Antibacterial Group**	ATC code	ATC Name (route[#])	Primary care	Secondary care	Broad-spectrum	AWaRe*	RTI
Tetracyclines	J01AA01	Demeclocycline				W	
	J01AA02	Doxycycline (O)	Y			A	
		Doxycycline (P)				A	

	J01AA04	Lymecycline				W	
	J01AA06	Oxytetracycline				W	
	J01AA07	Tetracycline				A	
	J01AA08	Minocycline				W	
	J01AA12	Tigecycline				R	
Amphenicols	J01BA01	Chloramphenicol				W	
Penicillin (excluding inhibitors)	J01CA01	Ampicillin				A	
	J01CA04	Amoxicillin (O)				A	
		Amoxicillin (P)				A	Y
	J01CA08	Pivmecillinam				A	
	J01CA12	Piperacillin				W	
	J01CA17	Temocillin				W	
	J01CA51	Ampicillin, combinations				A	
	J01CE01	Benzylpenicillin				A	
	J01CE02	Phenoxymethylpenicillin				A	Y
	J01CF05	Flucloxacillin				A	
Penicillin (with inhibitor combinations)	J01CR02	Amoxicillin and beta-lactamase inhibitor (O)			Y	W	
		Amoxicillin and beta-lactamase inhibitor (P)				W	
	J01CR03	Ticarcillin and beta-lactamase inhibitor				W	
	J01CR05	Piperacillin and beta-lactamase inhibitor				W	Y
	J01CR50	Combinations of penicillins				O	
First-generation cephalosporins	J01DB01	Cefalexin			Y	W	
	J01DB04	Cefazolin			Y	W	
	J01DB05	Cefadroxil			Y	W	
	J01DB09	Cefradine			Y	W	

Second-generation cephalosporins	J01DC01	Cefoxitin			Y	W	
	J01DC02	Cefuroxime (O)			Y	W	
		Cefuroxime (P)				W	Y
	J01DC04	Cefaclor			Y	W	
Third-generation cephalosporins	J01DD01	Cefotaxime			Y	W	
	J01DD02	Ceftazidime			Y	W	Y
	J01DD04	Ceftriaxone			Y	W	Y
	J01DD08	Cefixime			Y	W	
	J01DD13	Cefpodoxime			Y	W	
	J01DD52	Ceftazidime and beta-lactamase inhibitor				R	Y
Fourth-generation cephalosporins	J01DE01	Cefepime				R	
Monobactams	J01DF01	Aztreonam				R	
Carbapenems	J01DH02	Meropenem				R	Y
	J01DH03	Ertapenem				R	
	J01DH04	Doripenem				R	
	J01DH51	Imipenem/cilastatin				R	
Fifth-generation cephalosporins	J01DI01	Ceftobiprole medocaril				R	
	J01DI02	Ceftaroline fosamil				R	
	J01DI54	Ceftolozane and beta-lactamase inhibitor				R	
Sulfonamides and trimethoprim	J01EA01	Trimethoprim				A	
	J01EB04	Sulfapyridine				O	
	J01EC02	Sulfadiazine				O	
	J01ED05	Sulfamethoxypyridazine				O	
	J01EE01	Co-trimoxazole				A	
Macrolides	J01FA01	Erythromycin		Y		W	
	J01FA02	Spiramycin		Y		W	
	J01FA09	Clarithromycin		Y		W	

	J01FA10	Azithromycin (O & P)		Y		W	
	J01FA15	Telithromycin		Y		W	
Lincosamides	J01FF01	Clindamycin				W	
Streptogramins	J01FG01	Pristinamycin				W	
	J01FG02	Quinupristin/dalfopristin				W	
Aminoglycosides	J01GB01	Tobramycin				W	
	J01GB03	Gentamicin				A	
	J01GB05	Neomycin				A	
	J01GB06	Amikacin				W	
Fluoroquinolones	J01MA01	Ofloxacin			Y	W	
	J01MA02	Ciprofloxacin			Y	W	
	J01MA06	Norfloxacin			Y	W	
	J01MA12	Levofloxacin (O & P)			Y	W	Y
	J01MA14	Moxifloxacin			Y	W	
Other quinolones	J01MB02	Nalidixic acid				W	
Glycopeptides	J01XA01	Vancomycin				W	Y
	J01XA02	Teicoplanin				W	Y
Polymyxins	J01XB01	Colistin				R	
Steroid antimicrobials	J01XC01	Fusidic acid				A	
Imidazole derivatives	J01XD01	Metronidazole				A	
	J01XD02	Tinidazole				N/A^	
Nitrofuran derivatives	J01XE01	Nitrofurantoin				A	
Other antibacterials	J01XX01	Fosfomycin				R	
	J01XX05	Methenamine				O	
	J01XX08	Linezolid				R	Y
	J01XX09	Daptomycin				R	
	J01XX11	Tedizolid				R	

Oral Metronidazole	P01AB01	Metronidazole				A	
Anti-*Clostridioides difficile* agents	A07AA09	Vancomycin				W	
	A07AA12	Fidaxomicin				W	

Route of administration: O = Oral, P = Parenteral

* England AWaRe index: Access, Watch, Reserve and Other

^ not included in AWaRe index

Figure S1. All broad-spectrum items prescribed per 1000 population in primary care, January 2015 – October 2020

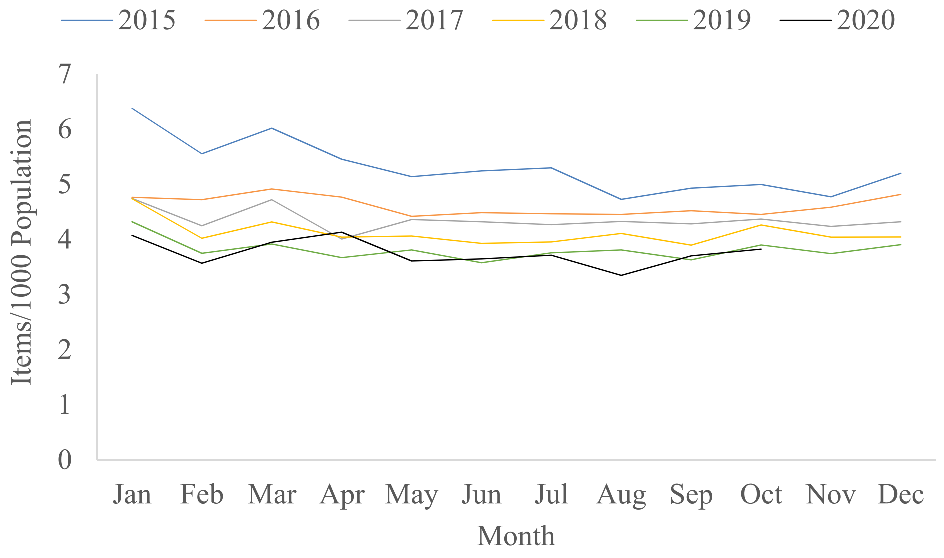

Broad-spectrum items include cephalosporins, fluoroquinolones and co-amoxiclav.

Figure S2. All antibacterial items for treatment of community-acquired pneumonia per 1000 population in primary care, January 2015 – October 2020

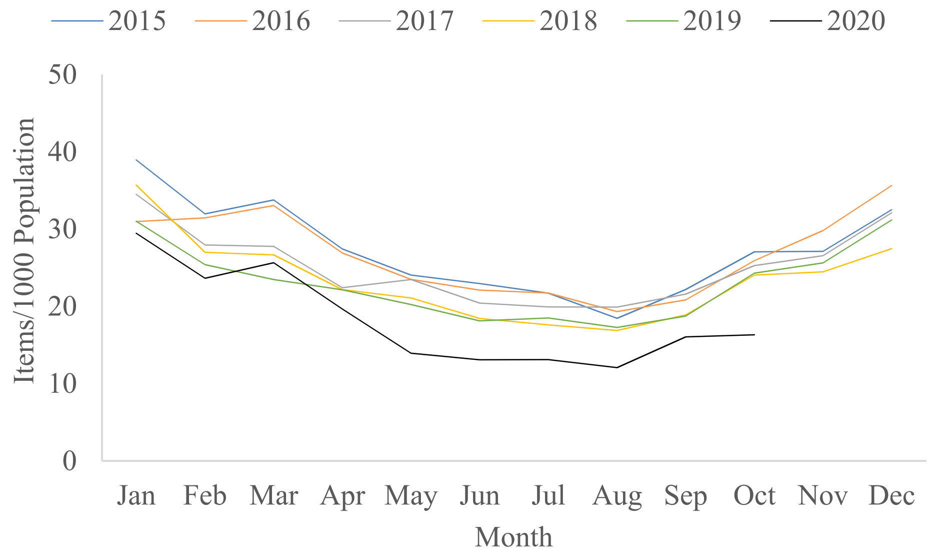

See list of antibacterial items for treatment of community-acquired pneumonia in Table S6.

Figure S3. First-line antibacterial items recommended for treatment of community-acquired pneumonia per 1000 population in primary care, January 2015 – October 2020

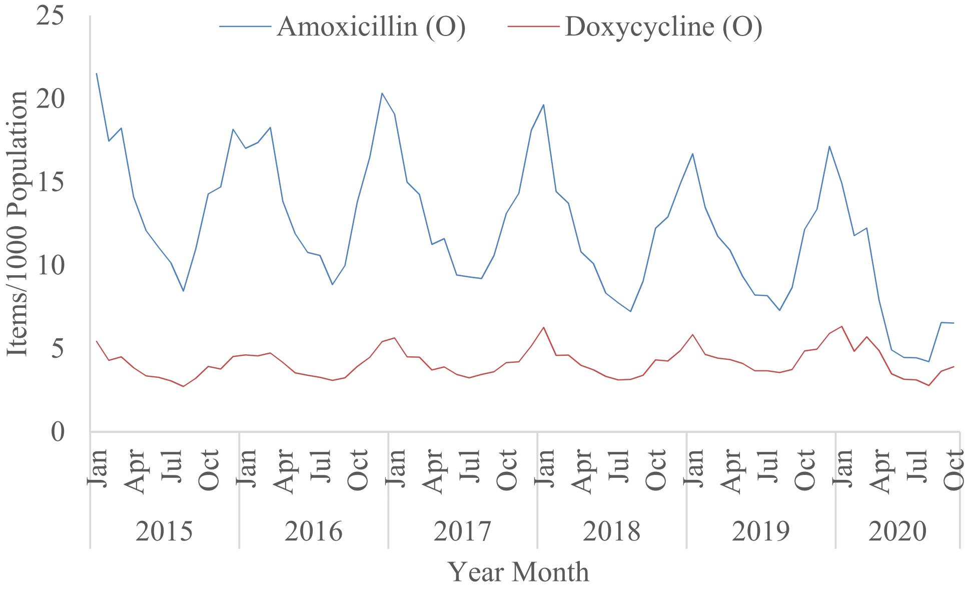

Figure S4. All antibacterial items for treatment of community-acquired pneumonia per 1000 population in primary care by age group, April 2015 – October 2020

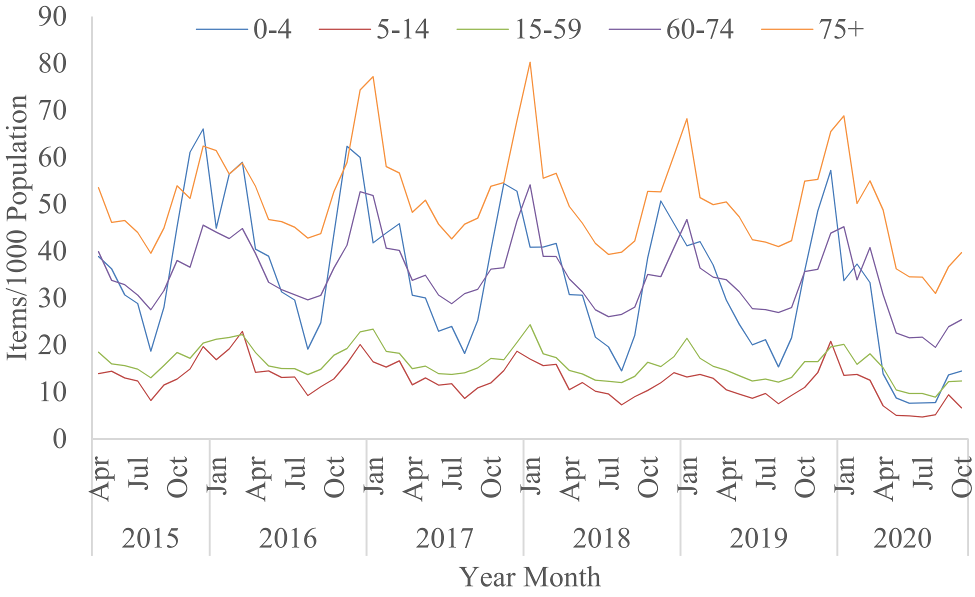

See list of antibacterial items for treatment of community-acquired pneumonia in Table S6.

Figure S5. All broad-spectrum items prescribed per 1000 population in primary care by age group, April 2015 – October 2020

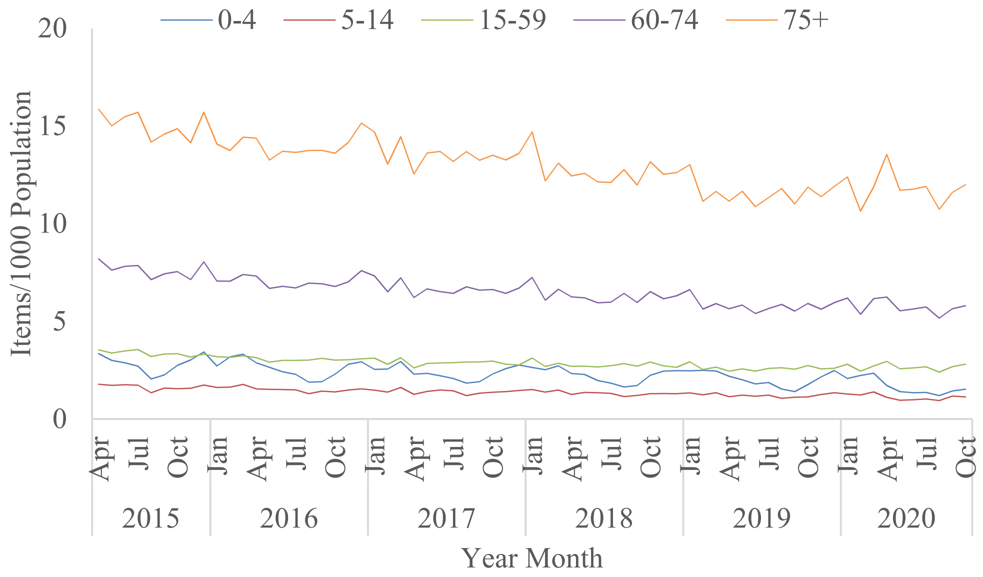

Broad-spectrum items include cephalosporins, fluoroquinolones and co-amoxiclav.

Figure S6. Co-amoxiclav items prescribed per 1000 population in primary care by age group, April 2015 – October 2020

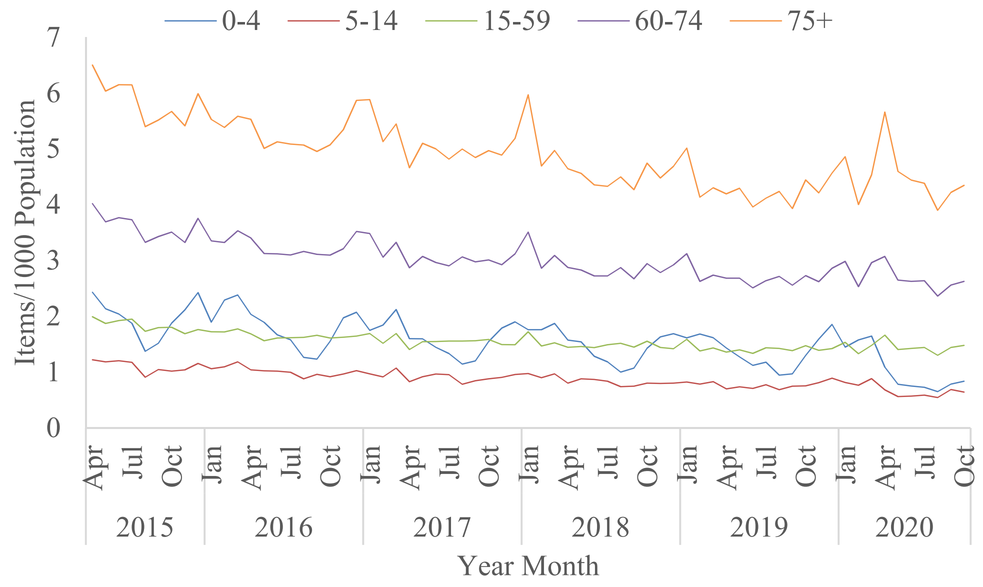

Figure S7. Oral amoxicillin items per 1000 population in primary care by age group, January 2015 – October 2020

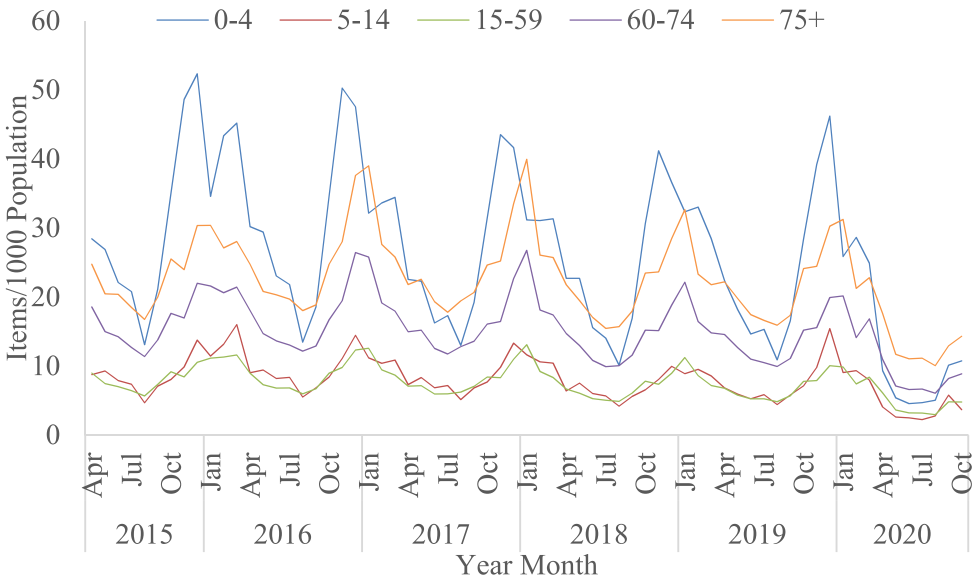

Figure S8. Oral amoxicillin and oral doxycycline use in DDDs per 1000 admissions in secondary care, January 2015 – October 2020

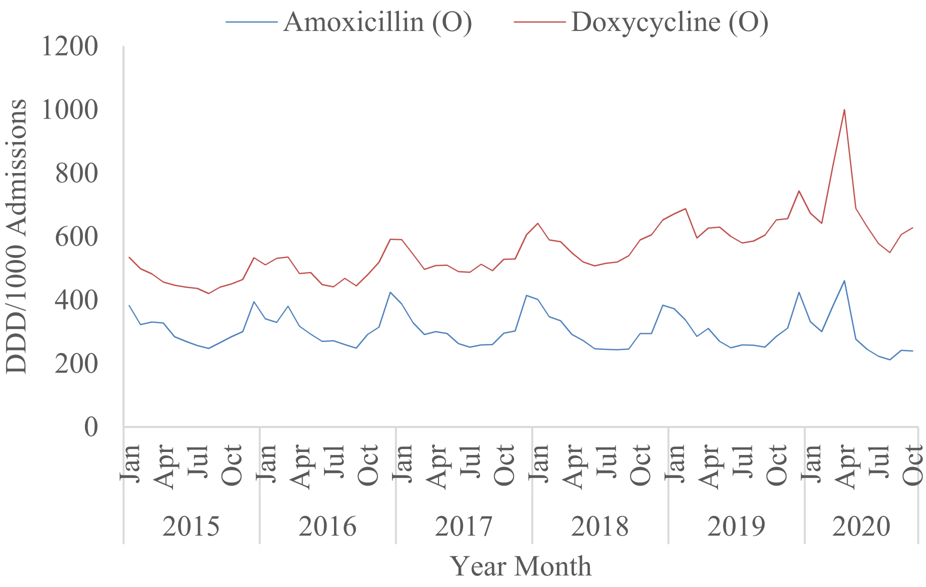

Figure S9. Third-generation cephalosporin use in DDDs per 1000 admissions in secondary care, January 2015 – October 2020

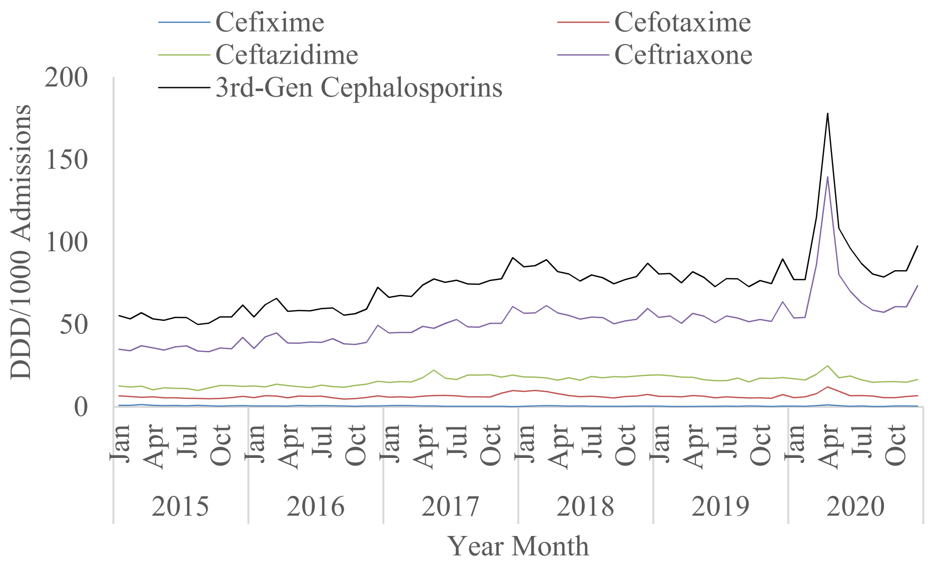

Figure S10. Macrolides use in DDDs per 1000 admissions in secondary care, January 2015 – October 2020

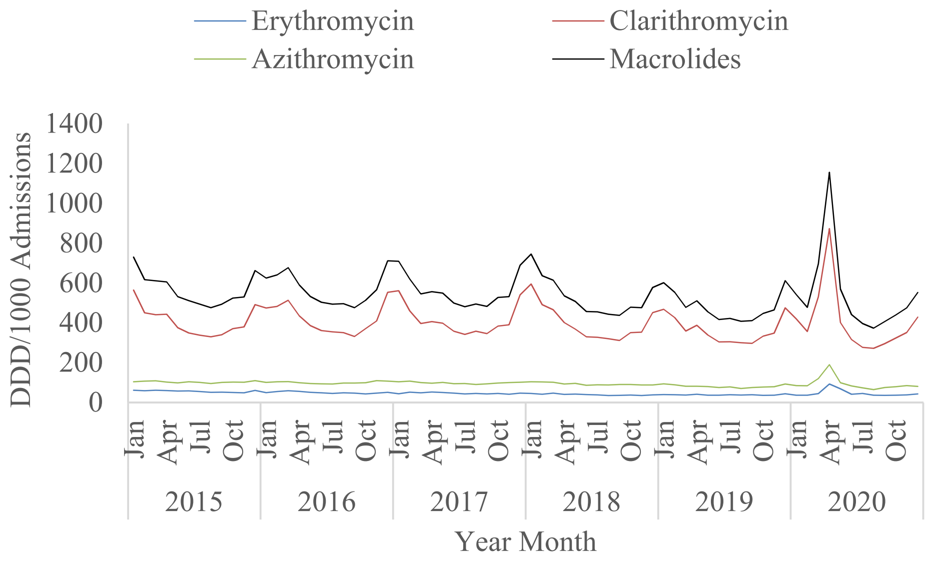

Figure S11. Piperacillin/tazobactam use in DDDs per 1000 admissions in secondary care, January 2015 – October 2020

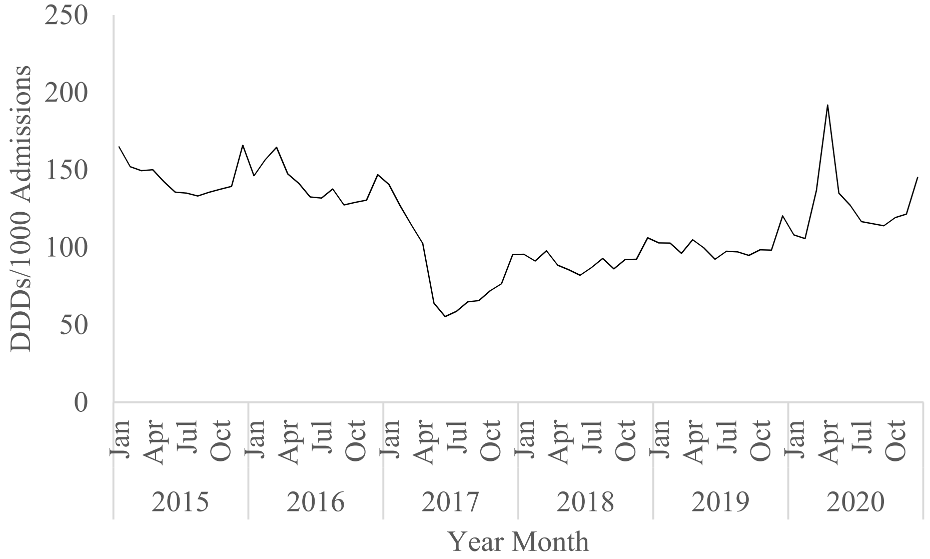

Figure S12. Carbapenems use in DDDs per 1000 admissions in secondary care by month, January 2015 – October 2020

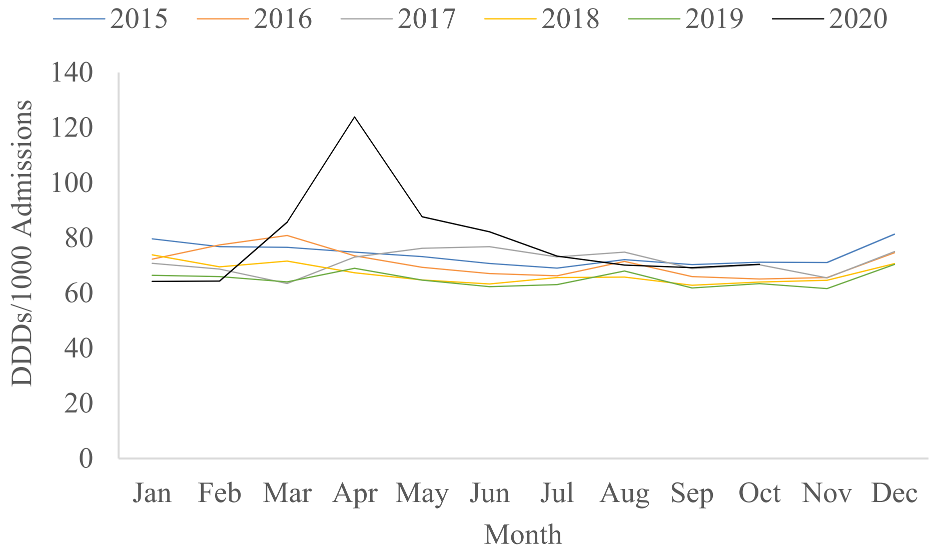

References

1. National Institute for Health and Care Excellence. COVID-19 rapid guideline: managing suspected or confirmed pneumonia in adults in the community. Available online: https://www.nice.org.uk/guidance/ng165/chapter/4-Managing-suspected-or-confirmed-pneumonia (accessed on 22 October 2020).
2. National Institute for Health and Care Excellence. Pneumonia (community-acquired): antimicrobial prescribing. Available online: https://www.nice.org.uk/guidance/ng138/chapter/Recommendations#choice-of-antibiotic (accessed on 22 October 2020).
3. National Institute for Health and Care Excellence. COVID-19 rapid guideline: antibiotics for pneumonia in adults in hospital. Available online: https://www.nice.org.uk/guidance/ng173 (accessed on 22 October 2020).
4. National Institute for Health and Care Excellence. Pneumonia (hospital-acquired): antimicrobial prescribing. Available online: https://www.nice.org.uk/guidance/ng139/chapter/Recommendations (accessed on 22 October 2020).
